# Crystal structures of 2,3-bis­(4-chloro­phen­yl)-1,3-thia­zolidin-4-one and *trans*-2,3-bis­(4-chloro­phen­yl)-1,3-thia­zolidin-4-one 1-oxide

**DOI:** 10.1107/S2056989015001954

**Published:** 2015-02-11

**Authors:** Hemant P. Yennawar, John Tierney, Patrick D. Hullihen, Lee J. Silverberg

**Affiliations:** a Department of Chemistry, Pennsylvania State University, University Park, PA 16802, USA; b Pennsylvania State University, Brandywine Campus, 312 M Main Building, 25, Yearsley Mill Rd, Media, PA 19063, USA; c Pennsylvania State University, Schuylkill Campus, 200 University Drive, Schuylkill Haven, PA 17972, USA

**Keywords:** crystal structure, thia­zolidine, thia­zolidin-4-one 1-oxide, hydrogen bonds, π–π inter­actions

## Abstract

In the crystal structures of the title compounds, C_15_H_11_Cl_2_NOS, (1), and C_15_H_11_Cl_2_NO_2_S, (2), wherein (2) is the oxidized form of (1), the thia­zolidine ring is attached to two chloro­phenyl rings. The chloro­phenyl ring on the 2-carbon atom position points in the same direction as that of the S atom in (1), while in (2), the S atom points in the opposite direction. The O atom on the chiral S atom in (2) is *trans* to the chloro­phenyl ring on the 2-carbon. The chloro­phenyl ring planes in each structure are close to orthogonal, making dihedral angles of 78.61 (6) and 87.46 (8)° in (1) and (2), respectively. The thia­zolidine ring has a twisted conformation on the S—C_methine_ bond in (1), and an envelope conformation with the S atom 0.715 (3) Å out of the plane of other four atoms in (2). In the crystal of (1), mol­ecules are linked by C—H⋯O hydrogen bonds, as well as by slipped parallel π–π inter­actions [inter-centroid distance = 3.840 (3) Å] between inversion-related phenyl rings, forming sheets parallel to (001). In the crystal of (2), mol­ecules are linked *via* C—H⋯O and C—H⋯Cl hydrogen bonds, forming slabs parallel to (001).

## Abstract 2

In the related title compounds, (1) and (2), the 3-thia­zolidine ring pucker is twisted on the S—C_methine_ bond in (1), while in (2), the ring has an envelope conformation with the S atom as the flap. In the crystal of (1), mol­ecules are linked by C—H⋯O hydrogen bonds forming chains along [100], while in the crystal of (2), mol­ecules are linked by C—H⋯O and C—H⋯Cl hydrogen bonds forming slabs parallel to (001).

## Chemical context   

1,3-Thia­zolidin-4-ones, also known as 4-thia­zolidinones, are known to have a wide range of biological activities (Jain *et al.*, 2012 ▸; Abhinit *et al.*, 2009 ▸; Hamama *et al.*, 2008 ▸; Singh *et al.*, 1981 ▸; Brown, 1961 ▸; Tripathi *et al.*, 2014 ▸; Prabhakar *et al.*, 2006 ▸). The *S*-oxides have been observed to show enhanced activity, for example, it was shown that on converting a 4-thia­zol­idinone to its sulfoxide and sulfone, the oxide showed greater activity against some cancer cell lines than the sulfide (Gududuru *et al.*, 2004 ▸). Oxidation from sulfide to sulfoxide makes the sulfur a chiral center, and produces *cis* and *trans* diastereomers with regard to the relationship of the oxygen atom attached to the S atom and the substituent at the 2-position (Rozwadowska *et al.*, 2002 ▸; Colombo *et al.*, 2008 ▸). The stereocenters may however be configurationally unstable in solution or even in the solid state (Rozwadowska *et al.*, 2002 ▸). We have previously reported on the preparation and NMR studies of a series of 2,3-diaryl-1,3-thia­zolidin-4-ones in which the two aryl groups had the same substitution pattern (Tierney *et al.*, 2005 ▸). In this study, we report on the *S*-oxidation of one of these compounds, 2,3-bis­(4-chloro­phen­yl)-1, 3-thia­zolidin-4-one (1), with Oxone (Trost & Curran, 1981 ▸; Yu *et al.*, 2012 ▸; Webb, 1994 ▸), which gave compound (2), and on their crystal structures.
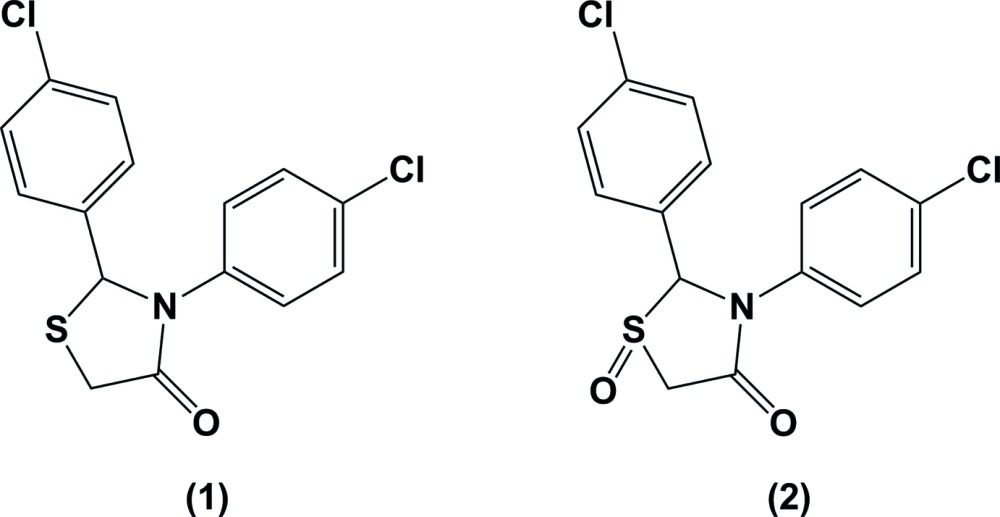


## Structural commentary   

**Figure 1:**
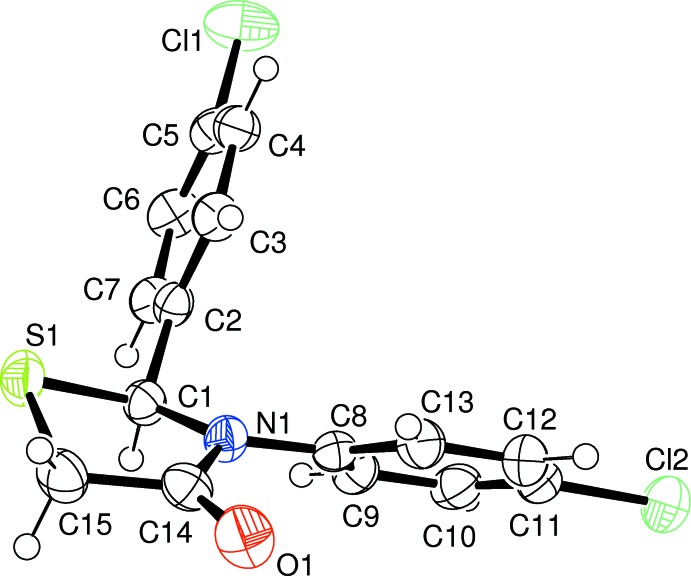
A view of the mol­ecular structure of compound (1), with atom labelling. Displacement ellipsoids are drawn at the 50% probability level.

The mol­ecular structures of compounds (1) and (2), Figs. 1 ▸ and 2 ▸, respectively, show a slight dissimilarity in the thia­zine ring conformation. In (1), the ring pucker is twisted on the S1—C1 bond, while in (2) the ring has an envelope conformation with atom S1 as the flap. The structures also differ in the disposition of the chloro­phenyl ring at atom C1. In (1), this ring points in the same direction as the S atom with respect to the thia­zolidine ring plane, while in (2), the S atom points in the opposite direction. The *trans* relationship between the oxygen atom on the S atom and the aromatic ring on C1 is favoured due to steric hindrance which would occur in the *cis* isomer. The chloro­phenyl rings are almost orthogonal to each other, making a dihedral angle of 78.61 (6)° in (1) and 87.46 (8)° in (2).

Comparison of the two structures shows that the oxygen–sulfur bond in (2) formed on the less hindered side of compound (1), away from the aryl group on C1, leading to a *trans* stereoisomer. Steric strain was further relieved by twisting so that both the aryl ring on C1 and the oxygen on S1 became pseudo-axial.

## Supra­molecular features   

**Figure 3:**
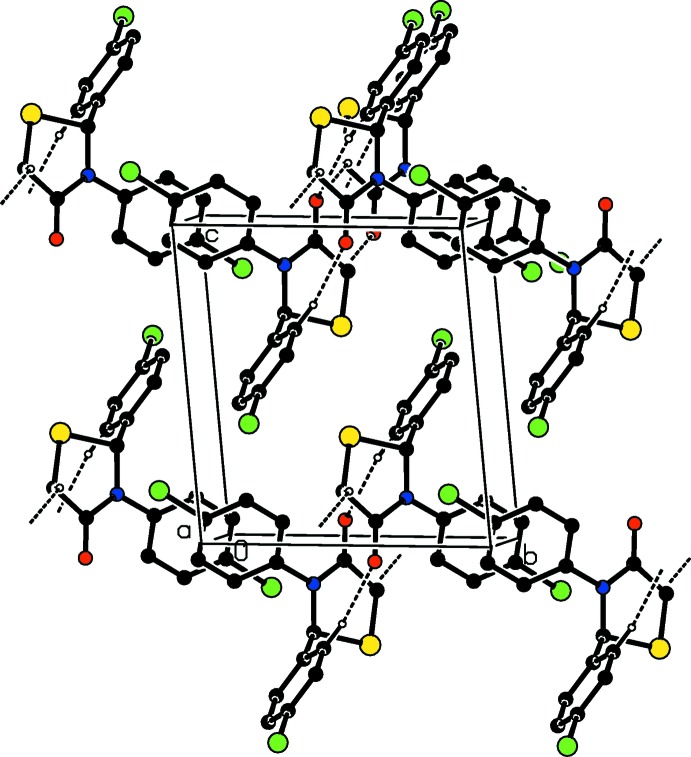
Crystal packing of compound (1) viewed along the *a* axis, showing the hydrogen bonds as dashed lines (see Table 1 ▸ for details; H atoms not involved in these inter­actions have been omitted for clarity).

In the crystal of (1), mol­ecules are linked *via* C—H⋯O hydrogen bonds, forming chains along [100]; see Table 1 ▸ and Fig. 3 ▸. The chains are linked *via* slipped parallel π–π inter­actions involving inversion-related chloro­phenyl rings, leading to the formation of sheets parallel to (001) [*Cg*3⋯*Cg*3^i^ = 3.840 (3) Å; *Cg*3 is the centroid of the C8–C13 ring; inter-planar distance = 3.3364 (7) Å; slippage = 1.901 Å; symmetry code: (i) −*x* + 2, −*y*, −*z* + 2].

**Figure 4:**
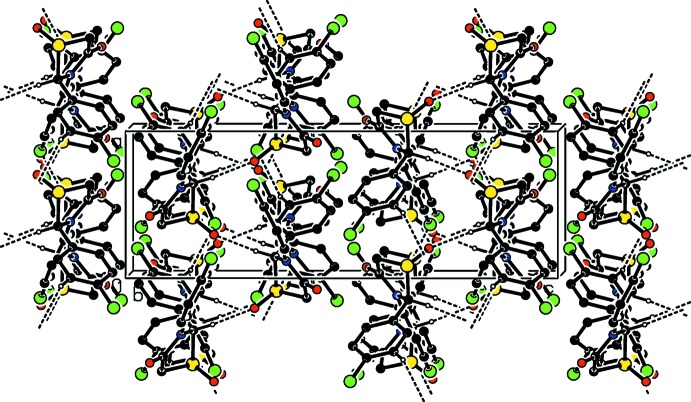
Crystal packing of compound (2) viewed along the *b* axis, showing the hydrogen bonds as dashed lines (see Table 2 ▸ for details; H atoms not involved in these inter­actions have been omitted for clarity).

In the crystal of (2), mol­ecules are linked *via* by C—H⋯O and C—H⋯Cl hydrogen bonds, forming slabs parallel to (001); see Table 2 ▸ and Fig. 4 ▸.

## Database survey   

Compound (1) differs from the previously reported 2,3-diphenyl-1, 3-thia­zolidin-4-one (Yennawar *et al.*, 2014 ▸) only in the presence of *p*-chlorine atoms on both phenyl rings, and the compound does not have a twist in the thia­zine ring. Compound (2) is related to 2-aryl-1,3-thia­zolidin-4-one 1-oxides, *viz*. 3-butyl-2-phenyl-1,3-thia­zolidine-1,4-dione (Wang *et al.*, 2010 ▸), (1*b*, 2*a*, 5*a*)-3, 5-dimethyl-1-oxo-2-phenyl-4-thia­zolidinone (Johnson *et al.*, 1983 ▸), 2-(2, 6-di­chloro­phen­yl)-3-(4, 5, 6-tri­methyl­pyrimidin-2-yl)-1, 3-thia­zolidin-4-one 1-oxide (Chen *et al.*, 2011 ▸) and *trans*-3-benzyl-2-(4-meth­oxy­phen­yl)thia­zolidin-4-one 1-oxide (Colombo *et al.*, 2008 ▸). All five compounds have a *trans* relationship between the O atom attached to the S atom and the 2-aryl ring.

## Synthesis and crystallization   

**Compound (1):** prepared as previously reported (Tierney *et al.*, 2005 ▸). Colourless block-like crystals were obtained by slow evaporation of a solution in ethanol.**Compound (2):** 2,3-bis (4-chloro­phen­yl)-1,3-thia­zolidin-4-one (1) (0.326 g, 1 mmol) was added to a 25 ml round-bottom flask. Methanol (4 ml) was added and the mixture was stirred at room temperature before cooling to 273–278 K. A solution of Oxone (0.456 g, 3.0 mmol calculated as KHSO_5_, 152.2 g mol^−1^) in distilled water (4 ml) was prepared. This solution (2.67 ml, 2 equivalents) was slowly added to the reaction mixture with stirring at 273–278 K. The reaction was followed by TLC. An additional aliquot of Oxone solution (0.67 ml) was added to convert the remaining starting material to sulfoxide. The mixture was extracted three times with methyl­ene chloride. The organic layers were combined and washed with water and saturated NaCl, then dried over sodium sulfate. The solution was concentrated under vacuum to give compound (2) as a crude solid. The solid was recrystallized from a mixture of methyl­ene chloride and hexane, and then dried (yield: 0.2413 g; 70.5%; m.p.: 406–409 K). Colourless plate-like crystals were obtained by slow evaporation of a solution in ethanol.

## Refinement details   

Crystal data, data collection and structure refinement details for structures (1) and (2) are summarized in Table 3 ▸. H atoms were positioned geometrically with C—H = 0.93–0.97 Å, and refined as riding with *U*_iso_(H) = 1.2*U*_eq_(C).

## Supplementary Material

Crystal structure: contains datablock(s) 1, 2, global. DOI: 10.1107/S2056989015001954/su5062sup1.cif

Structure factors: contains datablock(s) 1. DOI: 10.1107/S2056989015001954/su50621sup2.hkl

Structure factors: contains datablock(s) 2. DOI: 10.1107/S2056989015001954/su50622sup3.hkl

Supporting information file. DOI: 10.1107/S2056989015001954/su50621sup4.cml

Supporting information file. DOI: 10.1107/S2056989015001954/su50622sup5.cml

CCDC references: 1046346, 1046345

Additional supporting information:  crystallographic information; 3D view; checkCIF report

## Figures and Tables

**Figure 2:**
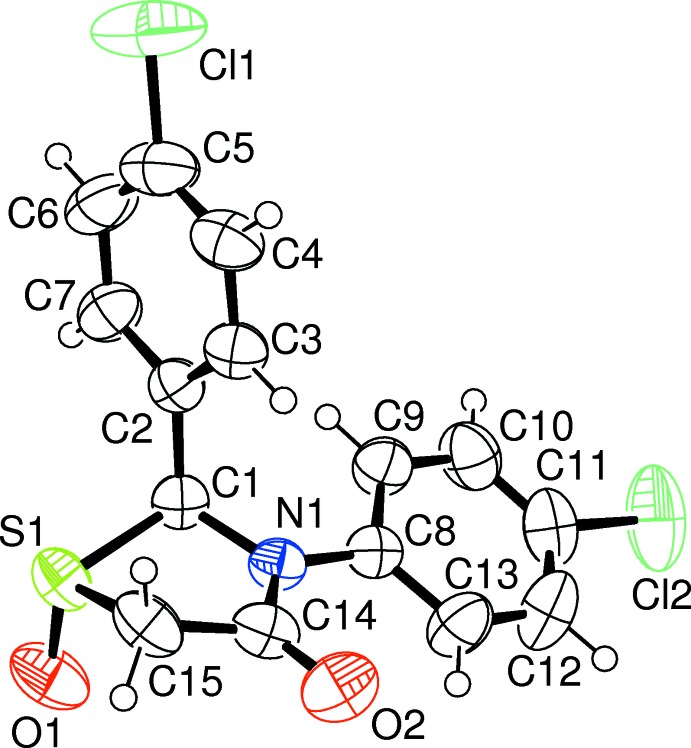
A view of the mol­ecular structure of compound (2), with atom labelling. Displacement ellipsoids are drawn at the 50% probability level.

**Table 1:** Hydrogen-bond geometry (Å, °) for (1)

*D*—H⋯*A*	*D*—H	H⋯*A*	*D*⋯*A*	*D*—H⋯*A*
C3—H3⋯O1^i^	0.93	2.48	3.326 (3)	151
C15—H15*B*⋯O1^ii^	0.97	2.46	3.221 (3)	135

Symmetry codes: (i) 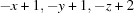; (ii) 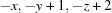.

**Table 2:** Hydrogen-bond geometry (Å, °) for (2)

*D*—H⋯*A*	*D*—H	H⋯*A*	*D*⋯*A*	*D*—H⋯*A*
C1—H1⋯O1^i^	0.98	2.19	3.154 (3)	167
C6—H6⋯Cl2^ii^	0.93	2.83	3.676 (3)	152

Symmetry codes: (i) 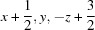; (ii) 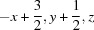.

**Table 3:** Experimental details

	(1)	(2)
Crystal data		
Chemical formula	C_15_H_11_Cl_2_NOS	C_15_H_11_Cl_2_NO_2_S
*M*_r_	324.21	340.21
Crystal system, space group	Triclinic, *P* 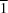	Orthorhombic, *P* *b* *c* *a*
Temperature (K)	298	298
*a*, *b*, *c* (Å)	8.019 (6), 9.562 (8), 9.984 (8)	7.1094 (17), 20.940 (5), 20.940
α, β, γ (°)	88.937 (13), 76.254 (12), 71.586 (13)	90, 90, 90
*V* (Å^3^)	704.3 (10)	3117.4 (11)
*Z*	2	8
Radiation type	Mo *K*α	Mo *K*α
μ (mm^−1^)	0.60	0.55
Crystal size (mm)	0.22 × 0.20 × 0.16	0.19 × 0.17 × 0.05

Data collection		
Diffractometer	Bruker *SMART* CCD area detector	Bruker *SMART* CCD area detector
Absorption correction	Multi-scan (*SADABS*; Bruker, 2001 ▸)	Multi-scan (*SADABS*; Bruker, 2001 ▸)
*T*_min_, *T*_max_	0.879, 0.910	0.902, 0.973
No. of measured, independent and observed [*I* > 2σ(*I*)] reflections	6575, 3406, 3070	26788, 3862, 2543
*R*_int_	0.016	0.038
(sin θ/λ)_max_ (Å^−1^)	0.666	0.666

Refinement		
*R*[*F* ^2^ > 2σ(*F* ^2^)], *wR*(*F* ^2^), *S*	0.036, 0.099, 1.05	0.051, 0.138, 1.07
No. of reflections	3406	3862
No. of parameters	181	190
H-atom treatment	H-atom parameters constrained	H-atom parameters constrained
Δρ_max_, Δρ_min_ (e Å^−3^)	0.24, −0.42	0.33, −0.31

Computer programs: *SMART* and *SAINT* (Bruker, 2001 ▸), *SHELXS97*, *SHELXL97* and *SHELXTL* (Sheldrick, 2008 ▸) and *PLATON* (Spek, 2009 ▸).

## supplementary crystallographic information

### (1) 2,3-Bis(4-chlorophenyl)-1,3-thiazolidin-4-one Crystal data

**Table:**

C_15_H_11_Cl_2_NOS	*Z* = 2
*M**_r_* = 324.21	*F*(000) = 332
Triclinic, *P*1	*D*_x_ = 1.529 Mg m^−^^3^
Hall symbol: -P 1	Mo *K*α radiation, λ = 0.71073 Å
*a* = 8.019 (6) Å	Cell parameters from 4305 reflections
*b* = 9.562 (8) Å	θ = 2.3–28.2°
*c* = 9.984 (8) Å	µ = 0.60 mm^−^^1^
α = 88.937 (13)°	*T* = 298 K
β = 76.254 (12)°	Block, colourless
γ = 71.586 (13)°	0.22 × 0.20 × 0.16 mm
*V* = 704.3 (10) Å^3^	

### (1) 2,3-Bis(4-chlorophenyl)-1,3-thiazolidin-4-one Data collection

**Table:**

Bruker SMART CCD area-detector diffractometer	3406 independent reflections
Radiation source: fine-focus sealed tube	3070 reflections with *I* > 2σ(*I*)
Graphite monochromator	*R*_int_ = 0.016
Detector resolution: 8.34 pixels mm^-1^	θ_max_ = 28.3°, θ_min_ = 2.1°
phi and ω scans	*h* = −10→10
Absorption correction: multi-scan (*SADABS*; Bruker, 2001)	*k* = −12→12
*T*_min_ = 0.879, *T*_max_ = 0.910	*l* = −13→13
6575 measured reflections	

### (1) 2,3-Bis(4-chlorophenyl)-1,3-thiazolidin-4-one Refinement

**Table:**

Refinement on *F*^2^	Primary atom site location: structure-invariant direct methods
Least-squares matrix: full	Secondary atom site location: difference Fourier map
*R*[*F*^2^ > 2σ(*F*^2^)] = 0.036	Hydrogen site location: inferred from neighbouring sites
*wR*(*F*^2^) = 0.099	H-atom parameters constrained
*S* = 1.05	*w* = 1/[σ^2^(*F*_o_^2^) + (0.0523*P*)^2^ + 0.2029*P*] where *P* = (*F*_o_^2^ + 2*F*_c_^2^)/3
3406 reflections	(Δ/σ)_max_ = 0.001
181 parameters	Δρ_max_ = 0.24 e Å^−^^3^
0 restraints	Δρ_min_ = −0.42 e Å^−^^3^

### (1) 2,3-Bis(4-chlorophenyl)-1,3-thiazolidin-4-one Special details

**Table:**

Experimental. The data collection nominally covered a full sphere of reciprocal space by a combination of 4 sets of ω scans each set at different φ and/or 2θ angles and each scan (10 s exposure) covering -0.300° degrees in ω. The crystal to detector distance was 5.82 cm.
Geometry. All e.s.d.'s (except the e.s.d. in the dihedral angle between two l.s. planes) are estimated using the full covariance matrix. The cell e.s.d.'s are taken into account individually in the estimation of e.s.d.'s in distances, angles and torsion angles; correlations between e.s.d.'s in cell parameters are only used when they are defined by crystal symmetry. An approximate (isotropic) treatment of cell e.s.d.'s is used for estimating e.s.d.'s involving l.s. planes.
Refinement. Refinement of *F*^2^ against ALL reflections. The weighted *R*-factor *wR* and goodness of fit *S* are based on *F*^2^, conventional *R*-factors *R* are based on *F*, with *F* set to zero for negative *F*^2^. The threshold expression of *F*^2^ > σ(*F*^2^) is used only for calculating *R*-factors(gt) *etc*. and is not relevant to the choice of reflections for refinement. *R*-factors based on *F*^2^ are statistically about twice as large as those based on *F*, and *R*- factors based on ALL data will be even larger.

### (1) 2,3-Bis(4-chlorophenyl)-1,3-thiazolidin-4-one Fractional atomic coordinates and isotropic or equivalent isotropic displacement parameters (Å^2^)

**Table:**

	*x*	*y*	*z*	*U*_iso_*/*U*_eq_	
C1	0.48888 (17)	0.30999 (15)	0.71043 (13)	0.0330 (3)	
H1	0.4491	0.2300	0.6838	0.040*	
C2	0.68202 (17)	0.28648 (14)	0.63246 (13)	0.0310 (3)	
C3	0.78163 (19)	0.36815 (16)	0.66932 (14)	0.0356 (3)	
H3	0.7306	0.4355	0.7455	0.043*	
C4	0.95613 (19)	0.34994 (16)	0.59348 (14)	0.0375 (3)	
H4	1.0233	0.4040	0.6184	0.045*	
C5	1.02943 (19)	0.24998 (16)	0.47982 (15)	0.0391 (3)	
C6	0.9333 (2)	0.16883 (17)	0.44035 (15)	0.0418 (3)	
H6	0.9841	0.1029	0.3631	0.050*	
C7	0.7584 (2)	0.18715 (16)	0.51810 (14)	0.0377 (3)	
H7	0.6920	0.1323	0.4932	0.045*	
C8	0.56811 (17)	0.21103 (15)	0.93089 (13)	0.0317 (3)	
C9	0.64973 (19)	0.06770 (15)	0.87310 (14)	0.0366 (3)	
H9	0.6364	0.0441	0.7872	0.044*	
C10	0.7510 (2)	−0.04056 (17)	0.94240 (16)	0.0422 (3)	
H10	0.8060	−0.1366	0.9034	0.051*	
C11	0.7694 (2)	−0.00403 (18)	1.07003 (17)	0.0434 (3)	
C12	0.6911 (2)	0.13821 (19)	1.12805 (16)	0.0457 (3)	
H12	0.7053	0.1614	1.2139	0.055*	
C13	0.5915 (2)	0.24636 (17)	1.05824 (15)	0.0395 (3)	
H13	0.5401	0.3429	1.0964	0.047*	
C14	0.29560 (18)	0.41916 (16)	0.93219 (15)	0.0364 (3)	
C15	0.19141 (19)	0.51206 (17)	0.83648 (16)	0.0429 (3)	
H15A	0.1473	0.6153	0.8689	0.051*	
H15B	0.0884	0.4811	0.8329	0.051*	
Cl1	1.25015 (6)	0.22688 (6)	0.38612 (5)	0.06617 (16)	
Cl2	0.89079 (7)	−0.14012 (6)	1.16121 (6)	0.06647 (16)	
N1	0.45860 (15)	0.32152 (13)	0.86178 (11)	0.0322 (2)	
O1	0.23825 (15)	0.42928 (15)	1.05662 (11)	0.0510 (3)	
S1	0.34401 (5)	0.48672 (5)	0.66875 (4)	0.04576 (12)	

### (1) 2,3-Bis(4-chlorophenyl)-1,3-thiazolidin-4-one Atomic displacement parameters (Å^2^)

**Table:**

	*U*^11^	*U*^22^	*U*^33^	*U*^12^	*U*^13^	*U*^23^
C1	0.0324 (6)	0.0375 (7)	0.0286 (6)	−0.0104 (5)	−0.0075 (5)	0.0019 (5)
C2	0.0327 (6)	0.0329 (6)	0.0252 (5)	−0.0080 (5)	−0.0064 (5)	0.0040 (5)
C3	0.0379 (7)	0.0386 (7)	0.0279 (6)	−0.0109 (5)	−0.0051 (5)	−0.0032 (5)
C4	0.0380 (7)	0.0413 (7)	0.0344 (7)	−0.0147 (6)	−0.0084 (5)	0.0018 (6)
C5	0.0354 (7)	0.0393 (7)	0.0352 (7)	−0.0084 (6)	0.0004 (5)	0.0025 (6)
C6	0.0465 (8)	0.0391 (7)	0.0328 (7)	−0.0108 (6)	0.0004 (6)	−0.0052 (6)
C7	0.0439 (7)	0.0372 (7)	0.0322 (6)	−0.0145 (6)	−0.0072 (6)	−0.0016 (5)
C8	0.0288 (6)	0.0361 (6)	0.0297 (6)	−0.0121 (5)	−0.0044 (5)	0.0056 (5)
C9	0.0371 (7)	0.0375 (7)	0.0333 (6)	−0.0120 (6)	−0.0053 (5)	0.0035 (5)
C10	0.0378 (7)	0.0370 (7)	0.0476 (8)	−0.0105 (6)	−0.0053 (6)	0.0093 (6)
C11	0.0367 (7)	0.0487 (8)	0.0499 (8)	−0.0184 (6)	−0.0153 (6)	0.0224 (7)
C12	0.0485 (8)	0.0583 (9)	0.0392 (7)	−0.0240 (7)	−0.0185 (6)	0.0127 (7)
C13	0.0413 (7)	0.0427 (7)	0.0368 (7)	−0.0155 (6)	−0.0110 (6)	0.0022 (6)
C14	0.0303 (6)	0.0402 (7)	0.0372 (7)	−0.0107 (5)	−0.0056 (5)	−0.0027 (5)
C15	0.0308 (6)	0.0451 (8)	0.0473 (8)	−0.0053 (6)	−0.0085 (6)	0.0014 (6)
Cl1	0.0465 (2)	0.0702 (3)	0.0689 (3)	−0.0232 (2)	0.0179 (2)	−0.0170 (2)
Cl2	0.0627 (3)	0.0667 (3)	0.0827 (3)	−0.0258 (2)	−0.0377 (3)	0.0424 (3)
N1	0.0307 (5)	0.0357 (5)	0.0269 (5)	−0.0076 (4)	−0.0050 (4)	0.0013 (4)
O1	0.0377 (5)	0.0675 (8)	0.0360 (5)	−0.0062 (5)	−0.0002 (4)	−0.0081 (5)
S1	0.0374 (2)	0.0520 (2)	0.0426 (2)	−0.00607 (16)	−0.01206 (16)	0.01364 (17)

### (1) 2,3-Bis(4-chlorophenyl)-1,3-thiazolidin-4-one Geometric parameters (Å, º)

**Table:**

C1—N1	1.473 (2)	C8—N1	1.4277 (18)
C1—C2	1.506 (2)	C9—C10	1.386 (2)
C1—S1	1.8282 (17)	C9—H9	0.9300
C1—H1	0.9800	C10—C11	1.380 (3)
C2—C7	1.386 (2)	C10—H10	0.9300
C2—C3	1.388 (2)	C11—C12	1.377 (3)
C3—C4	1.382 (2)	C11—Cl2	1.7455 (17)
C3—H3	0.9300	C12—C13	1.382 (2)
C4—C5	1.384 (2)	C12—H12	0.9300
C4—H4	0.9300	C13—H13	0.9300
C5—C6	1.373 (2)	C14—O1	1.212 (2)
C5—Cl1	1.7408 (19)	C14—N1	1.3751 (19)
C6—C7	1.390 (2)	C14—C15	1.510 (2)
C6—H6	0.9300	C15—S1	1.7930 (19)
C7—H7	0.9300	C15—H15A	0.9700
C8—C9	1.387 (2)	C15—H15B	0.9700
C8—C13	1.391 (2)		
			
N1—C1—C2	114.30 (11)	C10—C9—H9	119.8
N1—C1—S1	104.57 (9)	C8—C9—H9	119.8
C2—C1—S1	109.22 (10)	C11—C10—C9	119.14 (15)
N1—C1—H1	109.5	C11—C10—H10	120.4
C2—C1—H1	109.5	C9—C10—H10	120.4
S1—C1—H1	109.5	C12—C11—C10	121.07 (14)
C7—C2—C3	119.49 (13)	C12—C11—Cl2	119.13 (13)
C7—C2—C1	119.49 (12)	C10—C11—Cl2	119.80 (13)
C3—C2—C1	120.94 (12)	C11—C12—C13	119.72 (15)
C4—C3—C2	120.40 (13)	C11—C12—H12	120.1
C4—C3—H3	119.8	C13—C12—H12	120.1
C2—C3—H3	119.8	C12—C13—C8	120.07 (15)
C3—C4—C5	119.01 (13)	C12—C13—H13	120.0
C3—C4—H4	120.5	C8—C13—H13	120.0
C5—C4—H4	120.5	O1—C14—N1	124.72 (14)
C6—C5—C4	121.77 (14)	O1—C14—C15	122.94 (13)
C6—C5—Cl1	119.69 (12)	N1—C14—C15	112.33 (13)
C4—C5—Cl1	118.54 (12)	C14—C15—S1	107.22 (11)
C5—C6—C7	118.72 (14)	C14—C15—H15A	110.3
C5—C6—H6	120.6	S1—C15—H15A	110.3
C7—C6—H6	120.6	C14—C15—H15B	110.3
C2—C7—C6	120.60 (13)	S1—C15—H15B	110.3
C2—C7—H7	119.7	H15A—C15—H15B	108.5
C6—C7—H7	119.7	C14—N1—C8	121.42 (12)
C9—C8—C13	119.47 (13)	C14—N1—C1	115.85 (11)
C9—C8—N1	120.56 (13)	C8—N1—C1	120.65 (11)
C13—C8—N1	119.96 (13)	C15—S1—C1	91.77 (7)
C10—C9—C8	120.50 (14)		
			
N1—C1—C2—C7	138.56 (13)	C11—C12—C13—C8	1.0 (2)
S1—C1—C2—C7	−104.71 (14)	C9—C8—C13—C12	−1.8 (2)
N1—C1—C2—C3	−44.66 (17)	N1—C8—C13—C12	177.27 (13)
S1—C1—C2—C3	72.06 (15)	O1—C14—C15—S1	168.76 (13)
C7—C2—C3—C4	−0.5 (2)	N1—C14—C15—S1	−12.48 (15)
C1—C2—C3—C4	−177.31 (13)	O1—C14—N1—C8	6.5 (2)
C2—C3—C4—C5	0.5 (2)	C15—C14—N1—C8	−172.19 (12)
C3—C4—C5—C6	0.1 (2)	O1—C14—N1—C1	170.20 (14)
C3—C4—C5—Cl1	−179.42 (11)	C15—C14—N1—C1	−8.53 (17)
C4—C5—C6—C7	−0.7 (2)	C9—C8—N1—C14	136.45 (14)
Cl1—C5—C6—C7	178.88 (12)	C13—C8—N1—C14	−42.65 (18)
C3—C2—C7—C6	0.0 (2)	C9—C8—N1—C1	−26.44 (18)
C1—C2—C7—C6	176.81 (13)	C13—C8—N1—C1	154.46 (13)
C5—C6—C7—C2	0.6 (2)	C2—C1—N1—C14	144.10 (13)
C13—C8—C9—C10	1.3 (2)	S1—C1—N1—C14	24.72 (14)
N1—C8—C9—C10	−177.86 (12)	C2—C1—N1—C8	−52.09 (16)
C8—C9—C10—C11	0.2 (2)	S1—C1—N1—C8	−171.47 (9)
C9—C10—C11—C12	−1.0 (2)	C14—C15—S1—C1	22.62 (11)
C9—C10—C11—Cl2	178.08 (11)	N1—C1—S1—C15	−26.42 (10)
C10—C11—C12—C13	0.4 (2)	C2—C1—S1—C15	−149.16 (10)
Cl2—C11—C12—C13	−178.68 (12)		

### (1) 2,3-Bis(4-chlorophenyl)-1,3-thiazolidin-4-one Hydrogen-bond geometry (Å, º)

**Table:**

*D*—H···*A*	*D*—H	H···*A*	*D*···*A*	*D*—H···*A*
C3—H3···O1^i^	0.93	2.48	3.326 (3)	151
C15—H15*B*···O1^ii^	0.97	2.46	3.221 (3)	135

Symmetry codes: (i) −*x*+1, −*y*+1, −*z*+2; (ii) −*x*, −*y*+1, −*z*+2.

### (2) 2,3-Bis(4-chlorophenyl)-1,3-thiazolidin-4-one 1-oxide Crystal data

**Table:**

C_15_H_11_Cl_2_NO_2_S	*F*(000) = 1392
*M**_r_* = 340.21	*D*_x_ = 1.450 Mg m^−^^3^
Orthorhombic, *P**b**c**a*	Mo *K*α radiation, λ = 0.71073 Å
Hall symbol: -P 2ab 2ac	Cell parameters from 5771 reflections
*a* = 7.1094 (17) Å	θ = 2.2–28.2°
*b* = 20.940 (5) Å	µ = 0.55 mm^−^^1^
*c* = 20.940 Å	*T* = 298 K
*V* = 3117.4 (11) Å^3^	Plate, colourless
*Z* = 8	0.19 × 0.17 × 0.05 mm

### (2) 2,3-Bis(4-chlorophenyl)-1,3-thiazolidin-4-one 1-oxide Data collection

**Table:**

Bruker SMART CCD area-detector diffractometer	3862 independent reflections
Radiation source: fine-focus sealed tube	2543 reflections with *I* > 2σ(*I*)
Graphite monochromator	*R*_int_ = 0.038
Detector resolution: 8.34 pixels mm^-1^	θ_max_ = 28.3°, θ_min_ = 2.0°
phi and ω scans	*h* = −9→9
Absorption correction: multi-scan (*SADABS*; Bruker, 2001)	*k* = −27→27
*T*_min_ = 0.902, *T*_max_ = 0.973	*l* = −27→27
26788 measured reflections	

### (2) 2,3-Bis(4-chlorophenyl)-1,3-thiazolidin-4-one 1-oxide Refinement

**Table:**

Refinement on *F*^2^	Primary atom site location: structure-invariant direct methods
Least-squares matrix: full	Secondary atom site location: difference Fourier map
*R*[*F*^2^ > 2σ(*F*^2^)] = 0.051	Hydrogen site location: inferred from neighbouring sites
*wR*(*F*^2^) = 0.138	H-atom parameters constrained
*S* = 1.07	*w* = 1/[σ^2^(*F*_o_^2^) + (0.0581*P*)^2^ + 1.0427*P*] where *P* = (*F*_o_^2^ + 2*F*_c_^2^)/3
3862 reflections	(Δ/σ)_max_ = 0.003
190 parameters	Δρ_max_ = 0.33 e Å^−^^3^
0 restraints	Δρ_min_ = −0.31 e Å^−^^3^

### (2) 2,3-Bis(4-chlorophenyl)-1,3-thiazolidin-4-one 1-oxide Special details

**Table:**

Experimental. The data collection nominally covered a full sphere of reciprocal space by a combination of 4 sets of ω scans each set at different φ and/or 2θ angles and each scan (10 s exposure) covering -0.300° degrees in ω. The crystal to detector distance was 5.82 cm.
Geometry. All e.s.d.'s (except the e.s.d. in the dihedral angle between two l.s. planes) are estimated using the full covariance matrix. The cell e.s.d.'s are taken into account individually in the estimation of e.s.d.'s in distances, angles and torsion angles; correlations between e.s.d.'s in cell parameters are only used when they are defined by crystal symmetry. An approximate (isotropic) treatment of cell e.s.d.'s is used for estimating e.s.d.'s involving l.s. planes.
Refinement. Refinement of *F*^2^ against ALL reflections. The weighted *R*-factor *wR* and goodness of fit *S* are based on *F*^2^, conventional *R*-factors *R* are based on *F*, with *F* set to zero for negative *F*^2^. The threshold expression of *F*^2^ > σ(*F*^2^) is used only for calculating *R*-factors(gt) *etc*. and is not relevant to the choice of reflections for refinement. *R*-factors based on *F*^2^ are statistically about twice as large as those based on *F*, and *R*- factors based on ALL data will be even larger.

### (2) 2,3-Bis(4-chlorophenyl)-1,3-thiazolidin-4-one 1-oxide Fractional atomic coordinates and isotropic or equivalent isotropic displacement parameters (Å^2^)

**Table:**

	*x*	*y*	*z*	*U*_iso_*/*U*_eq_	
C1	0.1427 (3)	0.56601 (10)	0.84451 (10)	0.0447 (5)	
H1	0.1817	0.5615	0.7999	0.054*	
C2	0.2832 (3)	0.60780 (10)	0.87867 (10)	0.0446 (5)	
C3	0.3302 (3)	0.59815 (12)	0.94158 (11)	0.0508 (6)	
H3	0.2783	0.5640	0.9638	0.061*	
C4	0.4542 (4)	0.63897 (14)	0.97198 (12)	0.0627 (7)	
H4	0.4843	0.6326	1.0147	0.075*	
C5	0.5322 (4)	0.68858 (14)	0.93908 (15)	0.0710 (8)	
C6	0.4887 (4)	0.69891 (13)	0.87616 (16)	0.0745 (8)	
H6	0.5433	0.7327	0.8541	0.089*	
C7	0.3629 (4)	0.65874 (12)	0.84583 (12)	0.0594 (6)	
H7	0.3316	0.6658	0.8033	0.071*	
C8	0.2445 (3)	0.45232 (11)	0.85694 (9)	0.0455 (5)	
C9	0.4140 (3)	0.46695 (12)	0.82790 (12)	0.0563 (6)	
H9	0.4447	0.5094	0.8199	0.068*	
C10	0.5379 (4)	0.41909 (14)	0.81076 (13)	0.0684 (7)	
H10	0.6511	0.4293	0.7910	0.082*	
C11	0.4939 (5)	0.35700 (14)	0.82285 (13)	0.0716 (8)	
C12	0.3310 (5)	0.34158 (14)	0.85240 (14)	0.0808 (9)	
H12	0.3038	0.2990	0.8612	0.097*	
C13	0.2046 (4)	0.38873 (13)	0.86954 (13)	0.0677 (7)	
H13	0.0925	0.3778	0.8896	0.081*	
C14	−0.0326 (3)	0.49782 (12)	0.91318 (10)	0.0508 (6)	
C15	−0.1372 (3)	0.56016 (13)	0.91777 (10)	0.0593 (7)	
H15A	−0.0952	0.5839	0.9549	0.071*	
H15B	−0.2711	0.5523	0.9219	0.071*	
Cl1	0.68956 (15)	0.73930 (6)	0.97629 (6)	0.1258 (4)	
Cl2	0.64436 (17)	0.29545 (5)	0.79888 (5)	0.1196 (4)	
N1	0.1163 (2)	0.50246 (9)	0.87174 (8)	0.0434 (4)	
O1	−0.2071 (3)	0.57407 (11)	0.79699 (8)	0.0779 (6)	
O2	−0.0736 (3)	0.45046 (9)	0.94350 (8)	0.0715 (5)	
S1	−0.09086 (9)	0.60445 (3)	0.84674 (3)	0.0560 (2)	

### (2) 2,3-Bis(4-chlorophenyl)-1,3-thiazolidin-4-one 1-oxide Atomic displacement parameters (Å^2^)

**Table:**

	*U*^11^	*U*^22^	*U*^33^	*U*^12^	*U*^13^	*U*^23^
C1	0.0457 (12)	0.0505 (13)	0.0378 (10)	−0.0016 (10)	0.0072 (9)	0.0010 (9)
C2	0.0398 (11)	0.0484 (12)	0.0456 (11)	0.0011 (10)	0.0086 (9)	−0.0022 (9)
C3	0.0454 (13)	0.0594 (15)	0.0475 (12)	−0.0024 (11)	0.0063 (10)	−0.0046 (10)
C4	0.0507 (14)	0.0793 (19)	0.0580 (14)	−0.0008 (13)	0.0003 (12)	−0.0162 (13)
C5	0.0479 (15)	0.077 (2)	0.089 (2)	−0.0116 (14)	0.0071 (14)	−0.0282 (16)
C6	0.0671 (18)	0.0580 (17)	0.099 (2)	−0.0187 (14)	0.0212 (16)	−0.0015 (15)
C7	0.0607 (15)	0.0567 (15)	0.0607 (14)	−0.0082 (12)	0.0083 (12)	0.0027 (12)
C8	0.0469 (12)	0.0508 (13)	0.0387 (11)	−0.0038 (10)	0.0008 (9)	−0.0001 (9)
C9	0.0518 (14)	0.0577 (15)	0.0593 (14)	−0.0008 (11)	0.0120 (11)	0.0030 (11)
C10	0.0566 (16)	0.079 (2)	0.0700 (17)	0.0129 (14)	0.0092 (13)	0.0026 (14)
C11	0.080 (2)	0.0716 (19)	0.0636 (16)	0.0273 (16)	0.0024 (15)	0.0056 (14)
C12	0.110 (3)	0.0470 (15)	0.085 (2)	0.0075 (16)	0.0129 (19)	0.0078 (14)
C13	0.0747 (19)	0.0548 (16)	0.0735 (17)	−0.0092 (14)	0.0156 (14)	0.0053 (13)
C14	0.0445 (12)	0.0728 (16)	0.0352 (10)	−0.0074 (11)	0.0039 (9)	0.0008 (11)
C15	0.0434 (13)	0.0903 (19)	0.0442 (12)	0.0087 (13)	0.0042 (10)	−0.0051 (12)
Cl1	0.0974 (7)	0.1391 (9)	0.1409 (9)	−0.0599 (7)	−0.0018 (6)	−0.0500 (7)
Cl2	0.1392 (9)	0.1012 (7)	0.1184 (8)	0.0708 (7)	0.0144 (7)	0.0076 (6)
N1	0.0405 (9)	0.0512 (10)	0.0387 (8)	−0.0037 (8)	0.0068 (7)	0.0014 (8)
O1	0.0607 (12)	0.1197 (16)	0.0534 (10)	−0.0018 (11)	−0.0179 (9)	−0.0016 (10)
O2	0.0764 (13)	0.0803 (13)	0.0579 (10)	−0.0134 (10)	0.0236 (9)	0.0120 (9)
S1	0.0494 (4)	0.0702 (4)	0.0484 (3)	0.0080 (3)	−0.0057 (3)	0.0022 (3)

### (2) 2,3-Bis(4-chlorophenyl)-1,3-thiazolidin-4-one 1-oxide Geometric parameters (Å, º)

**Table:**

C1—N1	1.460 (3)	C8—N1	1.425 (3)
C1—C2	1.508 (3)	C9—C10	1.382 (4)
C1—S1	1.846 (2)	C9—H9	0.9300
C1—H1	0.9800	C10—C11	1.361 (4)
C2—C3	1.374 (3)	C10—H10	0.9300
C2—C7	1.390 (3)	C11—C12	1.352 (4)
C3—C4	1.383 (3)	C11—Cl2	1.749 (3)
C3—H3	0.9300	C12—C13	1.382 (4)
C4—C5	1.365 (4)	C12—H12	0.9300
C4—H4	0.9300	C13—H13	0.9300
C5—C6	1.371 (4)	C14—O2	1.213 (3)
C5—Cl1	1.728 (3)	C14—N1	1.372 (3)
C6—C7	1.382 (4)	C14—C15	1.506 (4)
C6—H6	0.9300	C15—S1	1.784 (2)
C7—H7	0.9300	C15—H15A	0.9700
C8—C9	1.384 (3)	C15—H15B	0.9700
C8—C13	1.387 (3)	O1—S1	1.4742 (19)
			
N1—C1—C2	115.38 (18)	C8—C9—H9	119.7
N1—C1—S1	105.78 (14)	C11—C10—C9	119.9 (3)
C2—C1—S1	109.30 (15)	C11—C10—H10	120.1
N1—C1—H1	108.7	C9—C10—H10	120.1
C2—C1—H1	108.7	C12—C11—C10	120.7 (3)
S1—C1—H1	108.7	C12—C11—Cl2	118.6 (2)
C3—C2—C7	119.2 (2)	C10—C11—Cl2	120.7 (2)
C3—C2—C1	122.0 (2)	C11—C12—C13	120.3 (3)
C7—C2—C1	118.7 (2)	C11—C12—H12	119.8
C2—C3—C4	120.4 (2)	C13—C12—H12	119.8
C2—C3—H3	119.8	C12—C13—C8	120.2 (3)
C4—C3—H3	119.8	C12—C13—H13	119.9
C5—C4—C3	119.8 (2)	C8—C13—H13	119.9
C5—C4—H4	120.1	O2—C14—N1	125.1 (2)
C3—C4—H4	120.1	O2—C14—C15	123.8 (2)
C4—C5—C6	120.9 (3)	N1—C14—C15	111.1 (2)
C4—C5—Cl1	120.2 (2)	C14—C15—S1	107.83 (15)
C6—C5—Cl1	118.9 (2)	C14—C15—H15A	110.1
C5—C6—C7	119.5 (3)	S1—C15—H15A	110.1
C5—C6—H6	120.3	C14—C15—H15B	110.1
C7—C6—H6	120.3	S1—C15—H15B	110.1
C6—C7—C2	120.2 (3)	H15A—C15—H15B	108.5
C6—C7—H7	119.9	C14—N1—C8	125.37 (19)
C2—C7—H7	119.9	C14—N1—C1	114.26 (19)
C9—C8—C13	118.3 (2)	C8—N1—C1	120.31 (17)
C9—C8—N1	119.3 (2)	O1—S1—C15	105.16 (13)
C13—C8—N1	122.4 (2)	O1—S1—C1	107.35 (11)
C10—C9—C8	120.6 (2)	C15—S1—C1	87.74 (10)
C10—C9—H9	119.7		
			
N1—C1—C2—C3	−23.1 (3)	C9—C8—C13—C12	−1.0 (4)
S1—C1—C2—C3	96.0 (2)	N1—C8—C13—C12	177.9 (2)
N1—C1—C2—C7	158.9 (2)	O2—C14—C15—S1	−158.4 (2)
S1—C1—C2—C7	−82.1 (2)	N1—C14—C15—S1	23.1 (2)
C7—C2—C3—C4	0.5 (3)	O2—C14—N1—C8	1.0 (4)
C1—C2—C3—C4	−177.5 (2)	C15—C14—N1—C8	179.50 (19)
C2—C3—C4—C5	−0.9 (4)	O2—C14—N1—C1	−176.0 (2)
C3—C4—C5—C6	0.4 (4)	C15—C14—N1—C1	2.4 (3)
C3—C4—C5—Cl1	−179.2 (2)	C9—C8—N1—C14	−163.5 (2)
C4—C5—C6—C7	0.5 (4)	C13—C8—N1—C14	17.7 (3)
Cl1—C5—C6—C7	−179.9 (2)	C9—C8—N1—C1	13.4 (3)
C5—C6—C7—C2	−0.8 (4)	C13—C8—N1—C1	−165.4 (2)
C3—C2—C7—C6	0.3 (4)	C2—C1—N1—C14	95.3 (2)
C1—C2—C7—C6	178.4 (2)	S1—C1—N1—C14	−25.7 (2)
C13—C8—C9—C10	1.4 (4)	C2—C1—N1—C8	−82.0 (2)
N1—C8—C9—C10	−177.5 (2)	S1—C1—N1—C8	157.09 (15)
C8—C9—C10—C11	−0.4 (4)	C14—C15—S1—O1	76.00 (19)
C9—C10—C11—C12	−1.0 (5)	C14—C15—S1—C1	−31.37 (17)
C9—C10—C11—Cl2	177.6 (2)	N1—C1—S1—O1	−72.87 (16)
C10—C11—C12—C13	1.4 (5)	C2—C1—S1—O1	162.31 (15)
Cl2—C11—C12—C13	−177.2 (2)	N1—C1—S1—C15	32.31 (16)
C11—C12—C13—C8	−0.4 (5)	C2—C1—S1—C15	−92.52 (16)

### (2) 2,3-Bis(4-chlorophenyl)-1,3-thiazolidin-4-one 1-oxide Hydrogen-bond geometry (Å, º)

**Table:**

*D*—H···*A*	*D*—H	H···*A*	*D*···*A*	*D*—H···*A*
C1—H1···O1^i^	0.98	2.19	3.154 (3)	167
C6—H6···Cl2^ii^	0.93	2.83	3.676 (3)	152

Symmetry codes: (i) *x*+1/2, *y*, −*z*+3/2; (ii) −*x*+3/2, *y*+1/2, *z*.
